# Influence of Environmental Fluctuations on Quantum Interference in Naphthalene and Azulene

**DOI:** 10.1002/smsc.202300075

**Published:** 2023-08-17

**Authors:** Jehan Alqahtani, Sara Sangtarash, Hatef Sadeghi

**Affiliations:** ^1^ Device Modelling Group School of Engineering University of Warwick CV4 7AL Coventry UK; ^2^ Department of Physics King Khalid University Abha 62529 Saudi Arabia

**Keywords:** environmental fluctuations, molecular electronics, naphthalene and azulene, quantum interference, quantum transport

## Abstract

Both naphthalene and azulene have the same number of carbon and hydrogen atoms, but the former is an alternant hydrocarbon and the latter is a nonalternant hydrocarbon. This leads to a large difference in their electronic and transport properties. Herein, quantum transport is investigated through these two molecules and it is shown how quantum interference (QI) affects their electrical conductance. It is demonstrated that the orbital rule to predict QI breaks down in both naphthalene and azulene. The influence of environmental fluctuations on their QI and electrical conductance is also investigated. The results show that QI in azulene is more sensitive to environmental fluctuations than in naphthalene. In particular, destructive QI can be changed to constructive QI in azulene by small environmental fluctuations.

## Graphical abstract

Environmental fluctuation have a large effect on electrical conductance and quantum interference (QI) in azulene single‐molecule junction. In particular, destructive QI changes to constructive QI in azulene by small environmental fluctuations. Furthermore, the orbital rule to predict QI breaks down in both naphthalene and azulene single molecules.

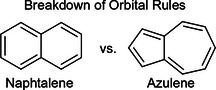

## 1 Introduction

Studying charge transport through single‐molecule junctions has helped us to develop our understanding of their electronic properties for nanoelectronic devices.^[^ ^1^, ^2^, ^3^ ^]^ Polyaromatic hydrocarbons (PAHs) such as benzene, naphthalene, and pyrene have attracted huge attention due to their simple but electronically rich structures.^[^ ^4^, ^5^, ^6^, ^7^, ^8^ ^]^ Among various PAH molecules, azulene has received a lot of attention in the field of single‐molecule electronics due to its unique structure and optical response.^[^ ^9^, ^10^, ^11^, ^12^ ^]^ It has the same number of hydrogens and carbons as naphthalene, but with five‐ (electron rich) and seven‐ (electron poor) membered rings. Therefore, it is considered to be a nonalternant hydrocarbon.^[^ ^13^ ^]^

There have been numerous theoretical and experimental investigations of electron transport through azulene.^[^ ^9^, ^10^, ^11^, ^12^, ^14^ ^]^ For example, conductance switching^[^ ^10^ ^]^ and relatively large tunnel magnetoresistance^[^ ^14^ ^]^ have been reported in this molecule. It has also been shown that the quantum interference (QI) rules can be challenged^[^ ^11^, ^15^, ^16^ ^]^ in azulene and QI can be controlled by protonation^[^ ^9^ ^]^ in this molecule. Despite these developments, there are still ambiguities in electronic properties of azulene. For example, azulene‐based junctions with constructive and destructive QI features show similar conductance.^[^ ^11^ ^]^ In the present work, we demonstrate that some of these ambiguities can originate from the sensitivity of QI in azulene to the environmental effect.^[^ ^17^, ^18^ ^]^

In this work, we investigate the electronic structure of azulene and its alternant hydrocarbon, naphthalene, and their transport properties between gold electrodes using first‐principle calculations. We study changes in QI through these molecules and demonstrate that the orbital rules that are widely used to predict QI break down in both molecules. We then develop a simple Hückel model to understand QI in these molecules. Finally, we examine the effect of environmental fluctuations on their quantum transport and QI properties and how it depends on the connection to the electrodes. We show that overall QI is more strongly influenced by these fluctuations in azulene than in naphthalene.

## 2 Result and Discussion

**Figure 1:**
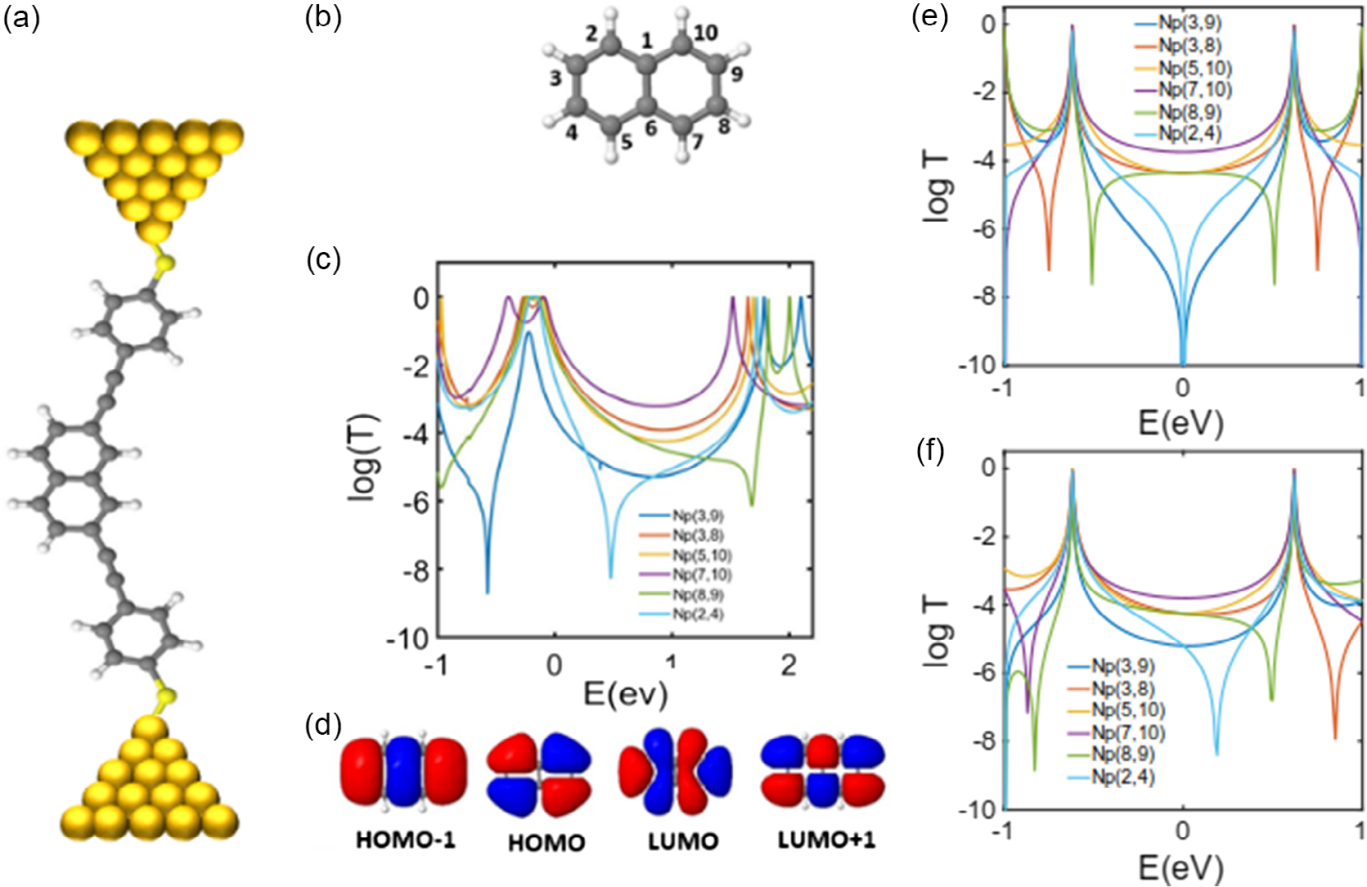
Quantum transport through naphthalene. a) Naphthalene core attached to gold electrodes via thiol anchor. b) Molecular structure of naphthalene with its numbering order. Note that the numbering convention used in this study does not correspond to the usual chemical numbering convention, but it is convenient theoretically and allows us to assign labels to all atoms. c) DFT transmission coefficient for naphthalene with different connectivities. d) Molecular orbital for naphthalene. e) A simple TB model when the on‐site energies *ε* are zero for all the sites and the coupling *γ* = −1 eV. f) Another TB model when ε of all sites are zero except sites 1 and 6 that are one and coupling is *γ* = −1 eV. In c, *E* = 0 eV denotes the DFT Fermi energy.

**Figure** **1**a shows the chemical structure of a single‐molecule junction consisting of a molecule with a naphthalene core connected to electrodes through thiol anchors. To study charge transport through these junctions, we first found the ground‐state geometries of junctions formed through different connection points (Figure 1b) using the SIESTA^[^ ^19^ ^]^ implementation of density functional theory (DFT). We then obtained the mean‐field Hamiltonian of each junction from DFT and combined it with our transport code GOLLUM^[^ ^20^, ^21^ ^]^ to calculate the transmission coefficient *T*(*E*) of electrons with energy *E* traversing from one gold electrode to the other (see Theoretical Methods section for more details).

Figure 1c shows *T*(*E*) for junctions with six different connection points to electrodes. In PAHs, destructive QI (DQI) is expected for molecules with odd‐to‐odd or even‐to‐even connection points to electrodes.^[^ ^22^ ^]^ For example, np(2,4) in Figure 1c shows an antiresonance which is characteristic of DQI. For all odd‐to‐even connectivities, constructive QI (CQI) is expected, for example, np(7,10) connectivity. Higher conductance is expected for connectivities with CQI features compared to those with DQI. This is indeed the case in naphthalene, where connectivities np(3,8), np(5,10), np(5,7), and np(8,9) show higher conductance than np(3,9) and np(2,4) around the DFT Fermi energy (*E* = 0 eV).

Despite these agreements, naphthalene also shows unexpected behavior for some connectivities. For example, np(3,9) does not show an antiresonance, while np(8,9) shows an antiresonance close to the lowest unoccupied molecular orbital (LUMO) resonance. From the orbital rule, this is not expected. From the orbital rules^[^ ^21^, ^23^ ^]^ (Equation (1)), a DQI is expected for np(3,9) and a CQI for np(8,9). The orbital rule states that DQI is expected if the sign of the products of wave functions for frontier orbitals (HOMO and LUMO) at connection points to electrodes is the same while CQI is expected if they were different.^[^ ^21^ ^]^ For example, if $\psi_{b}^{a}$ is the wavefunction at site *b* for state *a*, CQI (DQI) is expected if the signs of $\psi_{i}^{H}\psi_{j}^{H}$ and $\psi_{i}^{L}\psi_{j}^{L}$ are different (the same) where *H* and *L* denote HOMO and LUMO, respectively. This is because the transmission coefficient between sites *i* and *j* (*T*_*ij*_(*E*)) is proportional to the Green's function $g_{ij}$ of a molecule, defined as

(1) $$g_{ij}\left(E\right)\approx \frac{\psi_{i}^{H}\psi_{j}^{H}}{E-E_{H}}+\frac{\psi_{i}^{L}\psi_{j}^{L}}{E-E_{L}}$$

where *E*_a_ is the energy level associated with state *a*. From Equation (1), it is clear that $g_{ij}$ will only vanish (leading to an antiresonance and DQI) at a certain energy (e.g., *E *= (*E*_L_ *+ E*_H_)/2, the middle of the H–L gap^[^ ^24^ ^]^) if the signs of the products $\psi_{i}^{H}\psi_{j}^{H}$ and $\psi_{i}^{L}\psi_{j}^{L}$ are the same. For example, from Figure 1d, the sign of $\psi_{3}^{H}$ is positive (red colour) while $\psi_{9}^{H}$ is negative (blue color), thereby $\psi_{3}^{H}\psi_{9}^{H}<0$ for np(3,9). Similarly, $\psi_{3}^{L}\psi_{9}^{L}<0$. Therefore, from orbital rules, DQI is expected for np(3,9). Similarly, CQI is expected for np(8,9). These are not in agreement with the result obtained from the DFT transport calculations (Figure 1c) and demonstrate that orbital rule breaks down in naphthalene with np(3,9) and np(3,9) connection points to electrodes.

We found that this disagreement is due to the nonuniform charge distribution over the carbon atoms in naphthalene (Figure S3, Supporting Information). The charge on the central carbon atoms (sites 1 and 6 in Figure 1b) is different from that on the other sites. As a result, for np(8,9) connectivity, carbon atoms 2, 3, 4, and 5 act like a pendant group and create a Fano‐resonance^[^ ^21^, ^25^, ^26^ ^]^ close to the LUMO. For np(3,9), the antiresonance feature vanishes as a result of nonuniform charge distribution. Although there is no antiresonance feature in np(3,9), the overall low transmission is an indication of the effect of DQI for this connectivity.

In order to demonstrate this and further understand the surprising QI effect in np(3,9) and np(8,9), we built a one‐orbital‐per‐atom TB model, as shown in Figure 1e,f. If we treat all sites the same and set the on‐site energies to 0 eV and coupling integrals to −1 eV, the resulting transmissions for np(3,9) and np(8,9) are not in agreement with the DFT result (Figure 1c,e). However, by changing the on‐site energies at sites 1 and 6 to 1 eV (*ε*_1_ = *ε*_6_ = 1 eV), the DFT result is reproduced, as shown in Figure 1f. This supports our argument that the charge and therefore the potential energy on sites 1 and 6 are different from those at the other sites.

It is interesting to note that these changes have minimal effect on the connectivities with CQI feature. In what follows, we demonstrate that QI is more sensitive for some connectivities and influenced significantly with the environmental fluctuations. These fluctuations could be due to the presence of other molecules,^[^ ^2^, ^27^ ^]^ nearby ions^[^ ^28^ ^]^ or substrate,^[^ ^29^ ^]^ and residue from the junction fabrication process.^[^ ^30^ ^]^ In order to understand the effect of environmental fluctuations on QI through a naphthalene molecule, we constructed a TB model. We note that environmental fluctuations modify the potential profile through the molecule in the junction. To take this into account, we constructed a series of TB Hamiltonians by randomly changing the on‐site energies on the molecule, starting from the TB Hamiltonian that shows good agreement with our DFT result. We then calculate transmission coefficients through each junction (see Theoretical Methods section for details).

**Figure 2:**
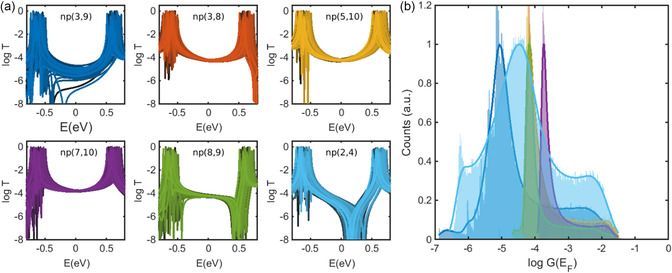
A TB model for naphthalene with various connectivities and on‐site energies. a) Individual TB transmission curves as a function of a random set of on‐site energies for different connectivities. b) Calculated conductance histograms for *T*(*E*)s in (a).

**Figure** **2**a shows the resulting transmissions for naphthalene with different connectivities. The position of the antiresonance for the np(3,9) connectivity is strongly affected by fluctuations on the on‐site energies, indicative of a great influence of environmental fluctuations on the QI pattern in this connectivity. Furthermore, the antiresonance features are more robust for np(2,4) and np(8,9). After applying a wide range of fluctuations (changing on‐site energies in the range of [−0.4, 0.4] eV), the antiresonance is clearly more resilient for np(2,4) and np(8,9). Between HOMO–LUMO gap, the transmissions are affected least in junctions with CQI features such as np(3,8), np(5,10), np(7,10). We then calculated room‐temperature electrical conductance using Landaure formula and then constructed computed histograms using the method discussed in our previous studies^[^ ^31^, ^32^ ^]^ (see also Theoretical Methods section for more details). Figure 2b shows the resulting histograms. The most probable conductance (histogram peak) is in good agreement with the result obtained from DFT (Figure 1c).

**Figure 3:**
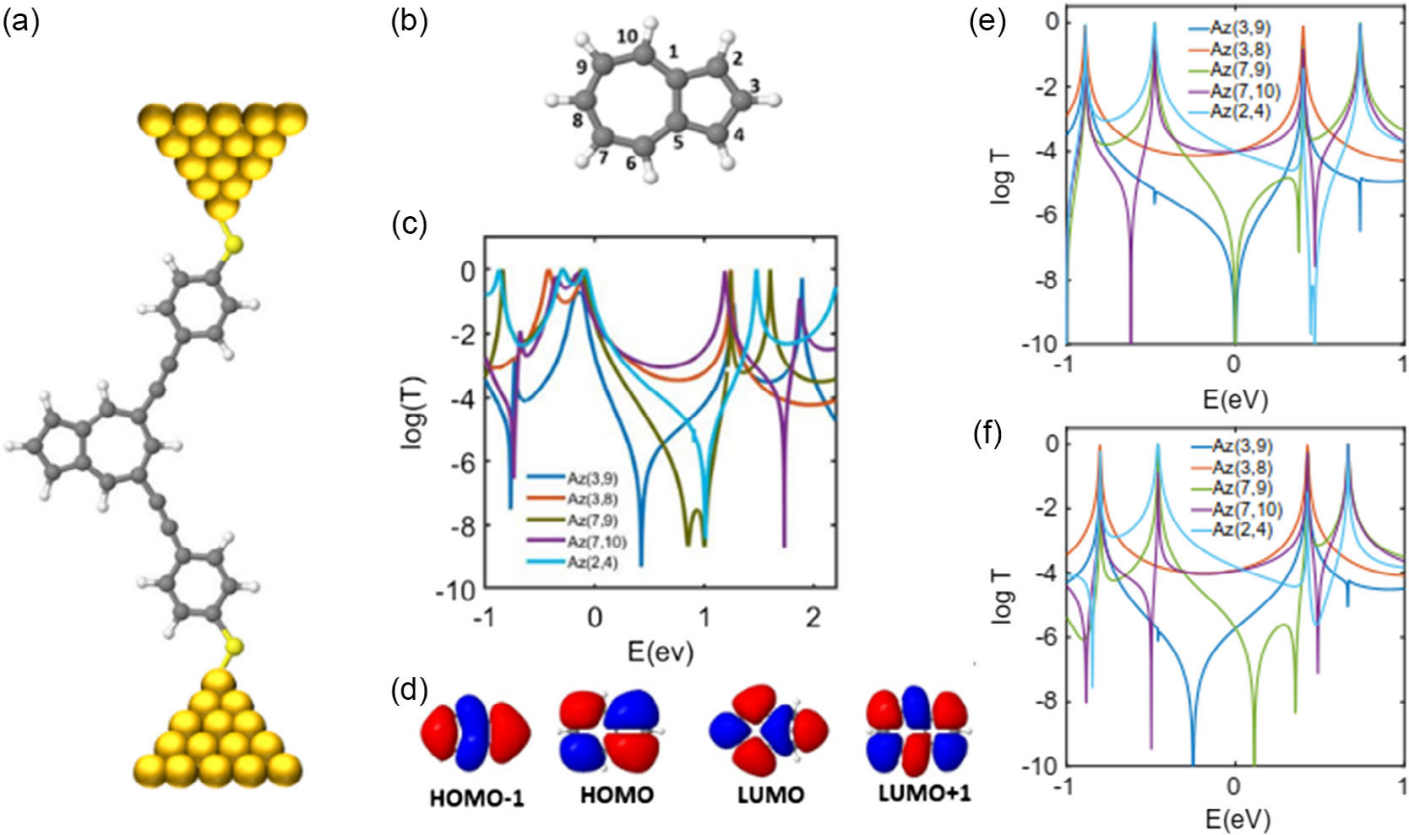
Quantum transport through azulene. a) Azulene core attached to gold electrodes via thiol anchor. b) Molecular structure of azulene with its numbering order. Note that the numbering convention used in this study does not correspond to the usual chemical numbering convention, but it is convenient theoretically and allows us to assign labels to all atoms. c) DFT transmission coefficient for azulene with different connectivities. d) Molecular orbital for azulene. e) Simple TB model when the on‐site energies ε are zero for all the sites and the coupling *γ* = −1 eV. f) Another model of TB when *ε* of all sites are zero except sites 1 and 5 that are 0.5 and sites 7, 8, and 9 that are −0.25 and the coupling is *γ* = −1 eV. In (c), *E* = 0 eV denotes the DFT Fermi energy.

Next, we consider the transport properties of azulene between metallic electrodes (**Figure** **3**a). Although naphthalene and azulene have the same number of carbons and hydrogens, there is a bond between sites 1 and 6 in naphthalene (Figure 1b), while atoms 1 and 5 are connected in azulene (Figure 3b). Therefore, azulene contains a negatively charged pentagon and positively charged heptagon.^[^ ^33^, ^34^, ^35^ ^]^ This results in a charge transfer between them, leading to a dipole moment. Also, the amplitude of the wavefunctions at sites 1 and 6 are zero for the HOMO and LUMO orbitals in naphthalene (Figure 1d). This is in contrast to azulene, where the amplitudes of the wavefunctions at sites 1 and 5 are nonzero for the HOMO and LUMO orbitals (Figure 3d). In what follows, we study quantum transport through azulene. Figure 3c shows the DFT transmission coefficients for azulene with different connection points to electrodes. First, all odd‐to‐odd or even‐to‐even connectivities show DQI, while all odd‐to‐even connectivities show CQI. This rule is generally expected to work in alternant hydrocarbons; however, it clearly works in azulene too. Nevertheless, the orbital rule does not work for az(2,4) connectivity. From the orbital rule, we expect a CQI in az(2,4); however, a DQI is predicted in DFT transmission. This is evidenced by a clear antiresonance feature in the DFT transmission for this connectivity.

In order to demonstrate the sensitivity of azulene to changes in charge distribution, we build a TB model using the same methodologies used for naphthalene. We first start with the simplest one‐orbital‐per‐atom TB model where all on‐site energies are set to 0 eV and all coupling integrals between the connected sites are set to −1 eV. This results in the transmission coefficients shown in Figure 3e which is not in qualitative agreement with our DFT result. To modify the model, we use the same approach as naphthalene and modify the on‐site energies based on the charge redistribution on the carbon atoms (Figure S3, Supporting Information). We found that by setting the on‐site energies of sites 1 and 5 to non‐zero values of 0.5 eV (sites that are connected) and site 7, 8, and 9 to −0.25 eV, our DFT and TB results show good qualitative agreement (Figure 3c,f).

**Figure 4:**
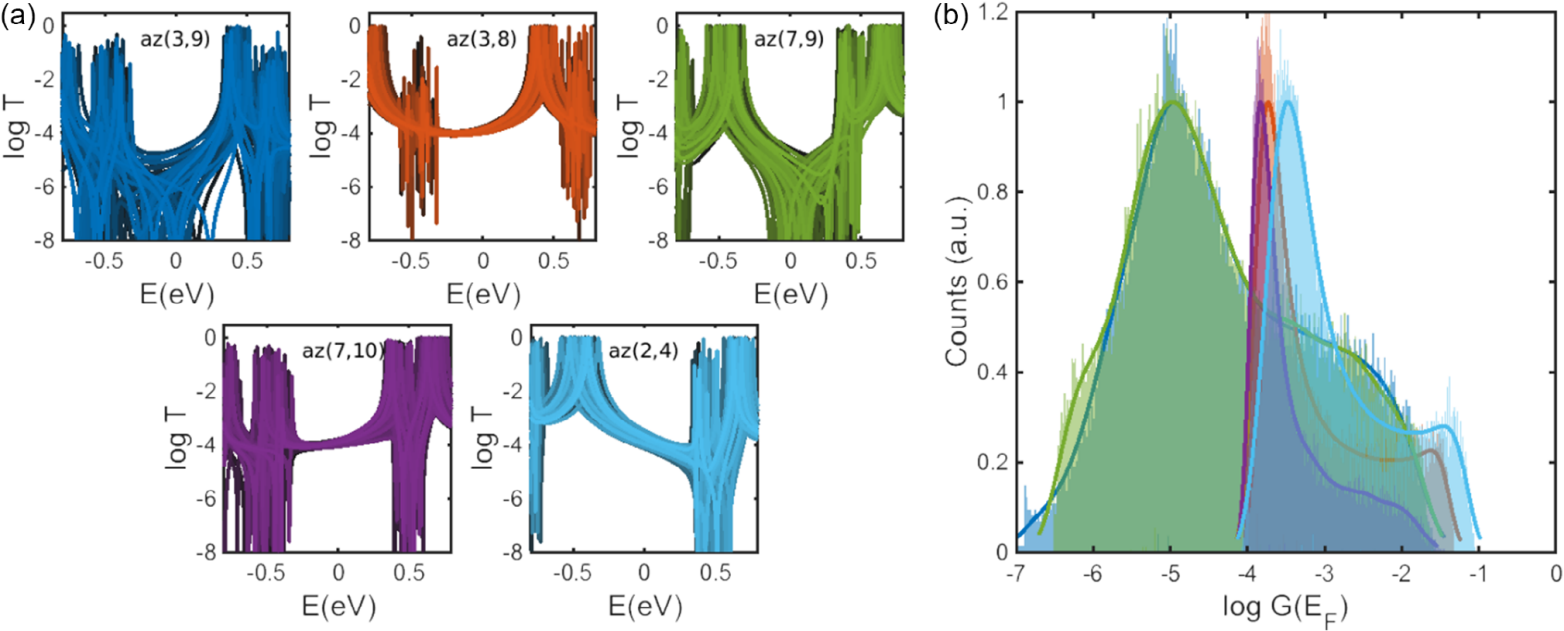
TB model for azulene with various connectivities and on‐site energies. a) Individual TB transmission curves as a function of a random set of on‐site energies for different connectivities. b) Calculated conductance histograms for *T*(*E*)s in (a).

In order to understand the effect of environmental fluctuations on QI through azulene, we calculate TB transmission coefficients by applying a random number in the range of [−0.4, 0.4] eV to the on‐site energies. We note that using a tip Au–S contact between the electrodes and molecules, we minimize the effect of fluctuations in the molecule–electrode conformations that in itself can lead to the variations in conductance.^[^ ^31^, ^36^ ^]^ This is because our aim is to demonstrate the role of fluctuations in the electronic structure of molecular cores on the charge transport efficiency of these junctions. **Figure** **4**a shows the resulting transmissions for each connectivity. The first notable difference between these results and the results obtained for naphthalene is that transmission through azulene is affected significantly for all connectivities with a DQI feature. For example, the antiresonance for az(3,9) and az(7,9) fluctuate strongly as a result of changes to the on‐site energies. Consequently, the antiresonance feature is expected to wash out in the ensemble‐averaged transmission for all connectivities. Figure 4b shows the computed conductance histograms obtained using the same methodology that we used for naphthalene. The most probable conductance is in remarkable agreement with the previous experimental results,^[^ ^9^, ^11^ ^]^ while it is not in agreement with the experiment reported in ref. [10] For example, in agreement with our theory, other studies^[^ ^9^, ^11^ ^]^ reported the conductance order of az(2,4) > az(7,10) > az(7,9) while the experimental result in ref. [10] shows az(7,10) > az(2,4). The agreement between our theory including fluctuations with the room‐temperature measurements studies in refs. [9, 11] and its disagreement with the low‐temperature measurement in ref. [10] suggest that the fluctuations play a significant role in determining the charge transport properties of azulene.

To show the evolution of changes in the QI pattern from naphthalene to azulene, we constructed a TB model, as shown in Figure S5–S11, Supporting Information. In this model, we start with a ring of 10 sites with on‐site energies *ε* = 0 eV and hopping integral *γ* = −1 eV. We then set the on‐site energies on sites 1 and 6 to 1 eV and the coupling between them to *γ* = −1 eV to create the naphthalene model. Next, we started reducing the coupling between sites 1 and 6, and at the same time, we increased the coupling between sites 1 and 5 by the same amount. We also decrease the on‐site energy at site 6 and increase the on‐site energy at site 5, 7, 8, and 9, as discussed in the azulene model above. With this approach, we can gradually move from the initial Hamiltonian of naphthalene to the final Hamiltonian of azulene. For each of these Hamiltonians, we calculated the transmission coefficient for different connectivities to the electrodes, as shown in Figure S5–S11, Supporting Information. We found that only for connectivities (3,9) and (8,9) do the interference pattern change from naphthalene to azulene (Figure S5 and S9, Supporting Information).

## 3 Conclusion

We have studied theoretically the electronic transport of naphthalene and azulene coupled to gold electrodes through thiol. We demonstrated the breakdown of orbital rules in naphthalene and azulene and showed that QI in both azulene and naphthalene is strongly dependent on environmental effects. We showed that QI in azulene is more sensitive to environmental fluctuations than in naphthalene. In particular, destructive QI can be changed to constructive QI in azulene by small fluctuations.

## 4 Theoretical Methods

4.1

4.1.1

The Hamiltonian of the structures described in this paper was obtained using DFT as described below or constructed from a simple TB model with a single orbital per atom as described in the maintext.

##### Density Functional Theory (DFT) Calculation

The optimized geometry and ground‐state Hamiltonian and overlap matrix elements of each structure were self‐consistently obtained using the SIESTA^[^ ^19^ ^]^ implementation of DFT. SIESTA uses norm‐conserving pseudopotentials to account for the core electrons and linear combinations of atomic orbitals to construct the valence states. The generalized gradient approximation (GGA) of the exchange and correlation functional was used with the Perdew–Burke–Ernzerhof parameterization,^[^ ^37^ ^]^ a double‐ζ polarized basis set, a real‐space grid defined with an equivalent energy cutoff of 250 Ry. The geometry optimization for each structure was performed for the forces smaller than 40 meV Å^−1^. The charge distribution in Figure S3, Supporting Information, is based on Mulliken charge distribution analysis implemented in the Gaussian g16 DFT code.^[^ ^38^ ^]^

##### Transport Calculation

The mean‐field Hamiltonian obtained from the converged DFT calculation or a simple TB Hamiltonian was combined with GOLLUM^[^ ^20^, ^21^ ^]^ implementation of the nonequilibrium Green's function method to calculate the phase‐coherent, elastic scattering properties of each system consisting of left (source) and right (drain) leads and the scattering region. The transmission coefficient T(E)for electrons of energy E (passing from the source to the drain) was calculated via the relation $T\left(E\right)=\text{trace}\left(\Gamma_{L}\left(E\right)G^{R}\left(E\right)\Gamma_{R}\left(E\right)G^{R^{†}}\left(E\right)\right)$. In this expression, $\Gamma_{L,R}\left(E\right)=i\left(\sum_{L,R}\left(E\right)-\sum_{L,R}^{†}\left(E\right)\right)$ describes the level broadening due to the coupling between left (L) and right (R) electrodes and the central scattering region, $\Sigma_{L,R}$(E) are the retarded self‐energies associated with this coupling, and $G_{R}={\left(ES-H-\Sigma_{L}-\Sigma_{R}\right)}^{-1}$ is the retarded Green's function, where *H* is the Hamiltonian and *S* is overlap matrix. Using the obtained transmission coefficient T(E), the conductance could be calculated by the Landauer formula^[^ ^39^ ^]^ $G=G_{0}\int dET\left(E\right)\left(-\frac{\partial f}{\partial E}\right)$ where $G_{0}=2e^{2}/h$ is the conductance quantum.

##### Conductance Histogram

To construct conductance histograms shown in Figure 2 and 4, we followed the same procedure as in our previous studies.^[^ ^31^, ^32^ ^]^ First, we formed a series of junctions with different TB Hamiltonians and calculated the electrical conductance *G* for a range of electrodes Fermi energies *E*_F_. Next, we created the conductance histograms using the calculated conductance for each junction and for a wide range of *E*_F_ between the frontier transport resonances. The peaks in the conductance histograms were fit with a log‐normal distribution and their center was defined as the most probable conductance.

## Conflict of Interest

The authors declare no conflict of interest.

## Supporting information

Supplementary Material

## Data Availability

All data in this paper can be reproduced using the method described in the paper. Data is also available on request from the authors.

## References

[1] N. Xin , J. Guan , C. Zhou , X. Chen , C. Gu , Y. Li , M. A. Ratner , A. Nitzan , J. F. Stoddart , X. Guo , Nat. Rev. Phys. 2019, 1, 211.

[2] F. Evers , R. Korytár , J. M. Van Ruitenbeek , Rev. Mod. Phys. 2020, 92, 35001.

[3] X. Xie , P. Li , Y. Xu , L. Zhou , Y. Yan , L. Xie , C. Jia , X. Guo , ACS Nano 2022, 16, 3476. 35179354 10.1021/acsnano.1c11433

[4] S. Sangtarash , C. Huang , H. Sadeghi , G. Sorohhov , J. Hauser , T. Wandlowski , W. Hong , S. Decurtins , S. X. Liu , C. J. Lambert , J. Am. Chem. Soc. 2015, 137, 11425. 26288219 10.1021/jacs.5b06558

[5] H. Sadeghi , J. Phys. Chem. C 2019, 123, 12556. 10.1021/acs.jpcc.8b12538 PMC7011773 32064012

[6] V. Kaliginedi , P. Moreno-Garcia , H. Valkenier , W. Hong , V. M. García-Suárez , P. Buiter , J. L. H. Otten , J. C. Hummelen , C. J. Lambert , T. Wandlowski , J. Am. Chem. Soc. 2012, 134, 5262. 22352944 10.1021/ja211555x

[7] J. Alqahtani , H. Sadeghi , S. Sangtarash , C. J. Lambert , Angew. Chem. Int. Ed. 2018, 57, 15065. 10.1002/anie.201807257 30208251

[8] Y. Geng , S. Sangtarash , C. Huang , H. Sadeghi , Y. Fu , W. Hong , T. Wandlowski , S. Decurtins , C. J. Lambert , S. X. Liu , J. Am. Chem. Soc. 2015, 137, 4469. 25781036 10.1021/jacs.5b00335

[9] G. Yang , S. Sangtarash , Z. Liu , X. Li , H. Sadeghi , Z. Tan , R. Li , J. Zheng , X. Dong , J. Liu , Y. Yang , J. Shi , Z. Xiao , G. Zhang , C. Lambert , W. Hong , D. Zhang , Chem. Sci. 2017, 8, 7505. 29163904 10.1039/c7sc01014a PMC5676185

[10] F. Schwarz , M. Koch , G. Kastlunger , H. Berke , R. Stadler , K. Venkatesan , E. Lçrtscher , E. Lörtscher , E. Lçrtscher , Angew. Chem., Int. Ed. 2016, 55, 11781. 10.1002/anie.201605559 27553767

[11] J. Xia , B. Capozzi , S. Wei , M. Strange , A. Batra , J. R. Moreno , R. J. Amir , E. Amir , G. C. Solomon , L. Venkataraman , L. M. Campos , Nano Lett. 2014, 14, 2941. 24745894 10.1021/nl5010702

[12] S. Sangtarash , H. Sadeghi , C. J. Lambert , Nanoscale 2016, 8, 13199. 27349309 10.1039/c6nr01907b

[13] H. N. Zeng , Z. M. Png , J. Xu , Chem. - Asian J. 2020, 15, 1904. 32333717 10.1002/asia.202000444

[14] Y. Matsuura , Phys. E (Amsterdam, Neth.) 2019, 105, 219.

[15] R. Stadler , Nano Lett. 2015, 15, 7175. 26485189 10.1021/acs.nanolett.5b03468

[16] M. Strange , G. C. Solomon , L. Venkataraman , L. M. Campos , Nano Lett. 2015, 15, 7177. 26485067 10.1021/acs.nanolett.5b04154

[17] S. Yuan , T. Gao , W. Cao , Z. Pan , J. Liu , J. Shi , W. Hong , Small Methods 2021, 5, 2001064. 10.1002/smtd.202001064 34927823

[18] Z. Huang , F. Chen , P. A. Bennett , N. Tao , J. Am. Chem. Soc. 2007, 129, 13225. 17915870 10.1021/ja074456t

[19] J. M. Soler , E. Artacho , J. D. Gale , A. García , J. Junquera , P. Ordejón , D. Sánchez-Portal , J. Phys.: Condens. Matter 2002, 14, 2745. 10.1088/0953-8984/20/6/064208 21693870

[20] J. Ferrer , C. J. Lambert , V. M. García-Suárez , D. Z. Manrique , D. Visontai , L. Oroszlany , R. Rodríguez-Ferradás , I. Grace , S. W. D. Bailey , K. Gillemot , H. Sadeghi , L. A. Algharagholy , New J. Phys. 2014, 16, 093029.

[21] H. Sadeghi , Nanotechnology 2018, 29, 373001. 29926808 10.1088/1361-6528/aace21

[22] S. Sangtarash , H. Sadeghi , C. J. Lambert , Phys. Chem. Chem. Phys. 2018, 20, 9630. 29578231 10.1039/c8cp00381e

[23] K. Yoshizawa , Acc. Chem. Res. 2012, 45, 1612. 22698647 10.1021/ar300075f

[24] S. Sangtarash , Ph.D. Thesis, Lancaster University, Lancaster, UK 2017.

[25] P. Gehring , H. Sadeghi , S. Sangtarash , C. S. Lau , J. Liu , A. Ardavan , J. H. Warner , C. J. Lambert , G. A. D. Briggs , J. A. Mol , C. Siong , P. Gehring , H. Sadeghi , S. Sangtarash , C. S. Lau , J. Liu , A. Ardavan , J. H. Warner , C. J. Lambert , G. A. D. Briggs , J. A. Mol , Nano Lett. 2016, 16, 4210. 27295198 10.1021/acs.nanolett.6b01104

[26] S. Sangtarash , H. Sadeghi , Nanoscale Adv. 2020, 2, 1031. 36133063 10.1039/c9na00649d PMC9418312

[27] D. Xiang , X. Wang , C. Jia , T. Lee , X. Guo , Chem. Rev. 2016, 116, 4318. 26979510 10.1021/acs.chemrev.5b00680

[28] H. Chen , J. Fraser Stoddart , Nat. Rev. Mater. 2021, 6, 804.

[29] J. Zhang , O. Braun , G. B. Barin , S. Sangtarash , J. Overbeck , R. Darawish , M. Stiefel , R. Furrer , A. Olziersky , K. Müllen , I. Shorubalko , A. H. S. Daaoub , P. Ruffieux , R. Fasel , H. Sadeghi , M. L. Perrin , M. Calame , Adv. Electron. Mater. 2023, 9, 2201204.

[30] P. Li , Y. Chen , B. Wang , M. Li , D. Xiang , C. Jia , X. Guo , Opto-Electronic Adv. 2022, 5, 210021.

[31] A. Daaoub , L. Ornago , D. Vogel , P. Bastante , S. Sangtarash , M. Parmeggiani , J. Kamer , N. Agraït , M. Mayor , H. van der Zant , H. Sadeghi , J. Phys. Chem. Lett. 2022, 13, 9156. 36166407 10.1021/acs.jpclett.2c01851 PMC9549519

[32] Y. Chelli , S. Sandhu , A. H. S. Daaoub , S. Sangtarash , H. Sadeghi , Nano Lett. 2023, 23, 3748. 37071608 10.1021/acs.nanolett.2c05068 PMC10176569

[33] S. V. Shevyakov , H. Li , R. Muthyala , A. E. Asato , J. C. Croney , D. M. Jameson , R. S. H. Liu , J. Phys. Chem. A 2003, 107, 3295.

[34] L. C. Murfin , S. E. Lewis , Molecules 2021, 26, 353.33445502

[35] L. Gai , J. Chen , Y. Zhao , J. Mack , H. Lu , Z. Shen , RSC Adv. 2016, 6, 32124.

[36] A. Saraiva-Souza , M. Smeu , H. Guo , Phys. Chem. Chem. Phys. 2020, 22, 3653. 32002522 10.1039/c9cp06159b

[37] J. P. Perdew , K. Burke , M. Ernzerhof , Phys. Rev. Lett. 1996, 77, 3865. 10062328 10.1103/PhysRevLett.77.3865

[38] M. J. Frisch , G. W. Trucks , H. B. Schlegel , G. E. Scuseria , M. A. Robb , J. R. Cheeseman , G. Scalmani , V. Barone , G. A. Petersson , H. Nakatsuji , X. Li , M. Caricato , A. V. Marenich , J. Bloino , B. G. Janesko , R. Gomperts , B. Mennucci , H. P. Hratchian , J. V. Ortiz , A. F. Izmaylov , J. L. Sonnenberg , Williams , F. Ding , F. Lipparini , F. Egidi , J. Goings , B. Peng , A. Petrone , T. Henderson , D. Ranasinghe , et al., Gaussian 16, Revision C.01, Gaussian Inc., Wallin gford, CT, USA 2016.

[39] R. Landauer , IBM J. Res. Dev. 1957, 1, 223.

