# Supplementary material for: Influence of Environmental Fluctuations on Quantum Interference in Naphthalene and Azulene
Source: Small Sci. 2023 Aug 17;3(10):2300075. doi: 10.1002/smsc.202300075 (PMC11935908; doi:10.1002/smsc.202300075)
Supplement: Supplementary file 1 — Supplementary Material [file SMSC-3-2300075-s001.pdf]

Supporting Information

Influence of Environmental Fluctuations on Quantum Interference in Naphthalene and Azulene

Jehan Alqahtani[1,2], Sara Sangtarash[1], and and Hatef Sadeghi[1,*]

[1] Device Modelling Group, School of Engineering, University of Warwick, CV4 7AL Coventry, UK
[2] Department of Physics, King Khalid University, Abha 62529, Saudi Arabia
Hatef.Sadeghi@warwick.ac.uk

Connectivity	Au-thiol-Naphthalene-thiol-Au
Np(3,9)	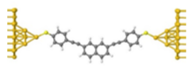
Np(3,8)	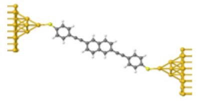
Np(5,10)	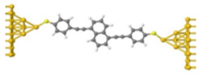
Np(7,10)	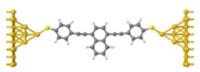
Np(8,9)	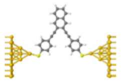
Np(2,4)	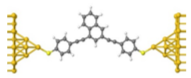

Figure S1. Molecular junctions studied in this paper formed by naphthalene connected to gold electrodes through thiol anchors.

Connectivity	Au-thiol-Azulene-thiol-Au
Az(3,9)	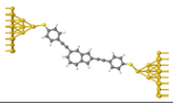
Az(3,8)	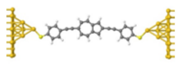
Az(7,9)	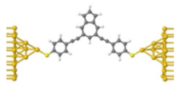
Az(7,10)	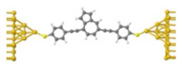
Az(2,4)	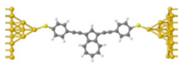

Figure S2. Molecular junctions studied in this paper formed by azulene connected to gold electrodes through thiol anchors.

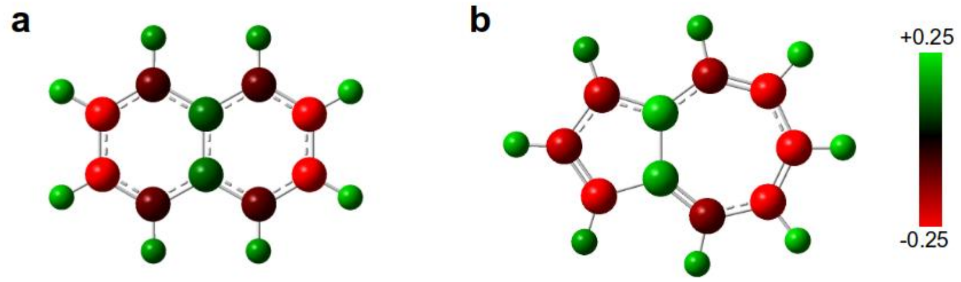

Figure S3. Mulliken charge distribution in (a) naphthalene, (b) azulene. Green and red colour represents positive and negative Δρ where Δρ is differences between the charge for a given atom before and after hybridisation. Red and green colours indicate negative and positive numbers, respectively.

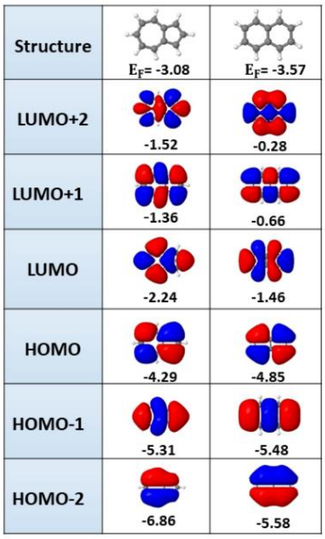

Figure S4. The DFT wavefunctions and energy levels of naphthalene and azulene.

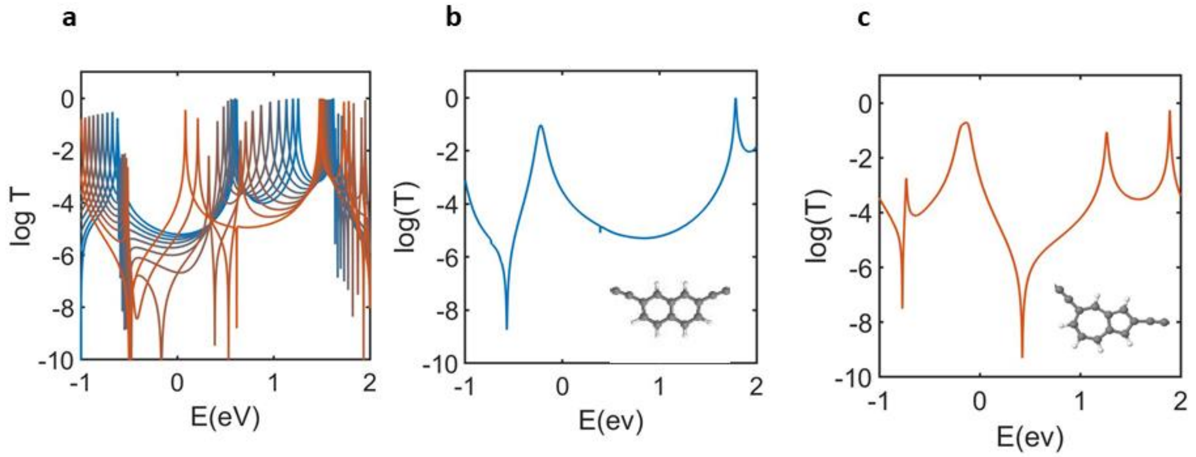

Figure S5. Transmission curves for (3, 9) connectivity. (a) A tight binding model of both naphthalene and azulene starting from blue (naphthalene) ending with red (azulene) – See the main text for the detail of methodology used. DFT result for (b) naphthalene and (c) azulene with the same connectivity.

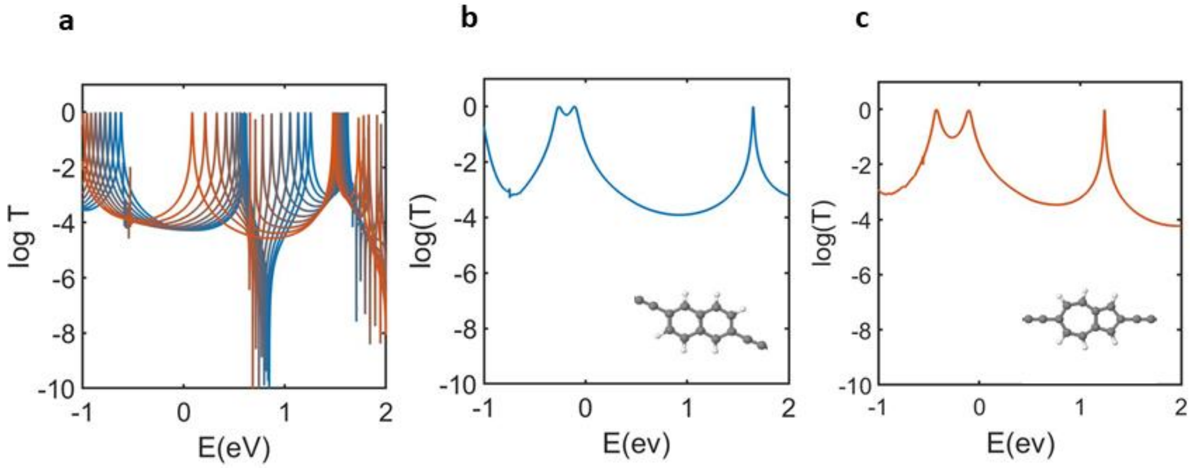

Figure S6. Transmission curves for (3, 8) connectivity. (a) A tight binding model of both naphthalene and azulene starting from blue (naphthalene) ending with red (azulene) – See the main text for the detail of methodology used. DFT result for (b) naphthalene and (c) azulene with the same connectivity.

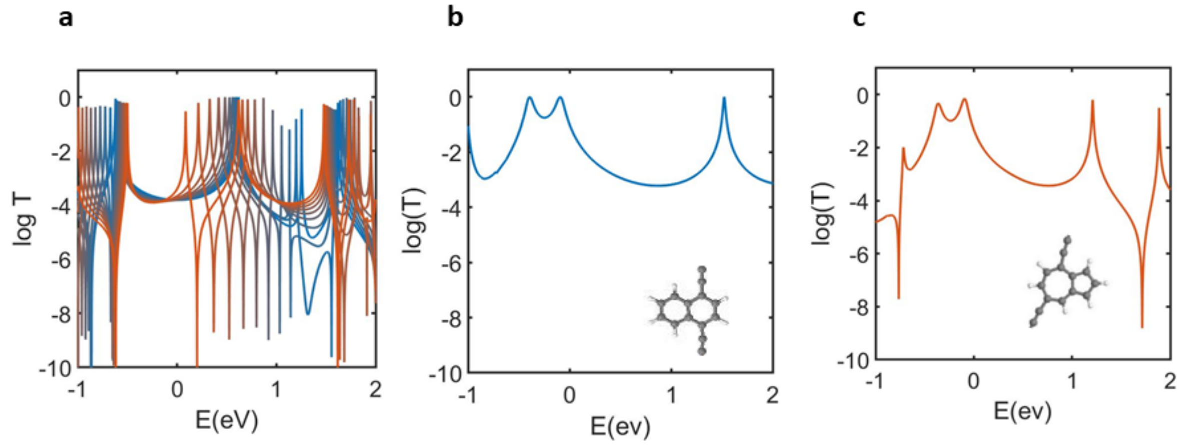

Figure S7. Transmission curves for site (7,10) connectivity. (a) A tight binding model of both naphthalene and azulene starting from blue (naphthalene) ending with red (azulene) – See the main text for the detail of methodology used. DFT result for (b) naphthalene and (c) azulene with the same connectivity.

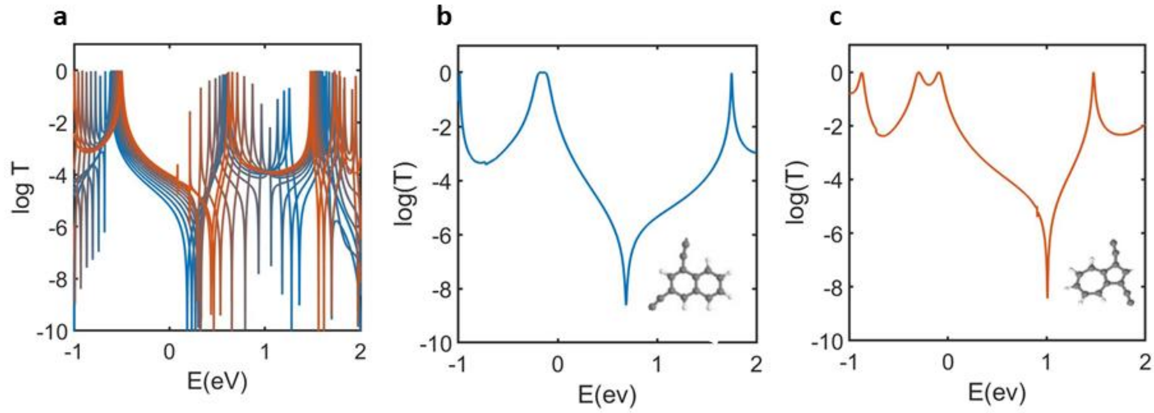

Figure S8. Transmission curves for site (2,4) connectivity. (a) A tight binding model of both naphthalene and azulene starting from blue (naphthalene) ending with red (azulene) – See the main text for the detail of methodology used. DFT result for (b) naphthalene and (c) azulene with the same connectivity.

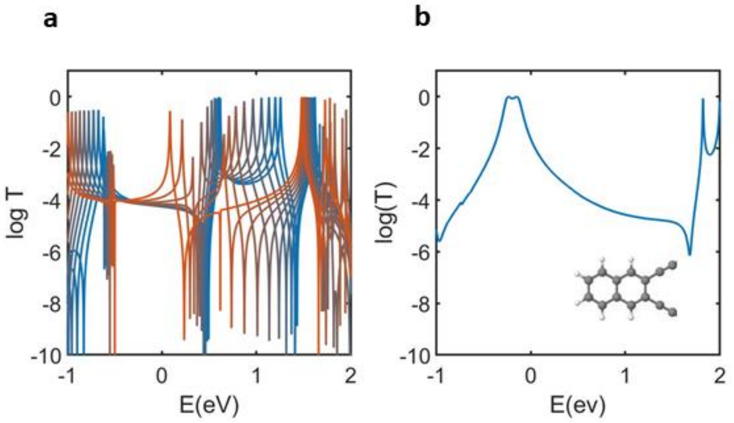

Figure S9. Transmission curves for site (8,9) connectivity. (a) A tight binding model of both naphthalene and azulene starting from blue (naphthalene) ending with red (azulene) – See the main text for the detail of methodology used. DFT result for (b) naphthalene with the same connectivity.

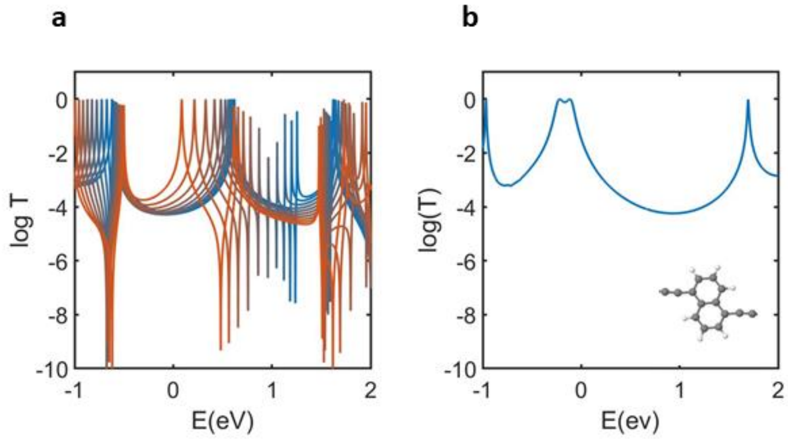

Figure S10. Transmission curves for site (5,10) connectivity. (a) A tight binding model of both naphthalene and azulene starting from blue (naphthalene) ending with red (azulene) – See the main text for the detail of methodology used. DFT result for (b) naphthalene with the same connectivity.

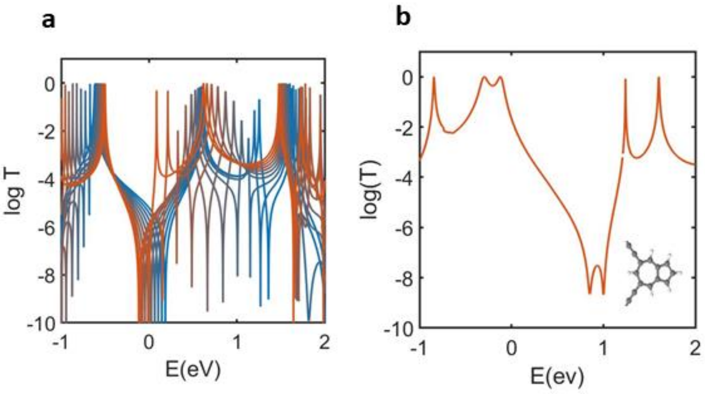

Figure S11. Transmission curves for site (6,9) connectivity. (a) A tight binding model of both naphthalene and azulene starting from blue (naphthalene) ending with red (azulene) – See the main text for the detail of methodology used. DFT result for (b) naphthalene with the same connectivity.

The detail of junction's constructions:

In order to construct the structures, we first find the optimised ground state geometry of gas phase molecules. We then connect the molecules to one electrode and find the relaxed ground state geometry of molecule bonded

to the electrode by fixing the gold atoms. We then perform final geometry optimisation after attaching the second electrode and find the ground state structure of the junction including the molecule between electrodes. After geometry optimisation between electrodes, the ground state Au-S-C distance and angle are 2.4Å and 119o, respectively in agreement with the previous studies[1]. The junction geometry therefore is affected by the structure of the molecule and depending on whether a molecule is bent or straight, the overall junction configuration will be different as in this study. However, the crucial point that can affect charge transfer is the Au-S-C distance and angle which is similar in all junctions.

References

[1] E. C. M. Ting, T. Popa, I. Paci, *Beilstein J. Nanotechnol.* **2016**, *7*, 53–61.
